# Influence of Thermal Inertia on Dynamic Characteristics of Gas Turbine Impeller Components

**DOI:** 10.3390/e27070711

**Published:** 2025-07-01

**Authors:** Yang Liu, Yuhao Jia, Yongbao Liu

**Affiliations:** College of Power Engineering, Naval University of Engineering, Wuhan 430033, China

**Keywords:** impeller components, thermal inertia, flow path heat transfer, dynamic characteristics

## Abstract

Gas turbines in land-based microgrids and shipboard-isolated power grids frequently face operational challenges, such as the startup and shutdown of high-power equipment and sudden load fluctuations, which significantly impact their performance. To examine the dynamic behavior of gas turbines under transitional operating conditions, a three-dimensional computational fluid dynamic simulation is employed to create a model of the gas turbine rotor, incorporating thermal inertia, which is then analyzed in conjunction with three-dimensional finite element methods. The governing equations of the flow field are discretized, providing results for the flow and temperature fields throughout the entire flow path. A hybrid approach, combining temperature differences and heat flux density, is applied to set the thermal boundary conditions for the walls, with the turbine’s operational state determined based on the direction of heat transfer. Additionally, mesh division techniques and turbulence models are selected based on the geometric dimensions and operating conditions of the compressor and turbine. The simulation results reveal that thermal inertia induces a shift in the dynamic characteristics of the rotor components. Under the same heat transfer conditions, variations in rotational speed have a minimal impact on the shift in the characteristic curve. The working fluid temperature inside the compressor components is lower, with a smaller temperature difference from the wall, resulting in less intense heat transfer compared to the turbine components. Overall, heat transfer accounts for only about 0.1% of the total enthalpy at the inlet. When heat exchange occurs between the working fluid and the walls, around 6–15% of the exchanged heat is converted into changes in technical work, with this percentage increasing as the temperature difference rises.

## 1. Introduction

As the temperature of the working fluid increases along the flow path, the temperature gradients between various components of the gas turbine, as well as between the turbine and the surrounding environment, become more pronounced [1]. This intensifies the impact of heat transfer on turbine performance. Moreover, modern naval gas turbines are affected by several factors, including uneven high temperatures in the combustion chamber [2], load fluctuations [3], cooling technologies, and thermal protection measures [4,5]. These factors create more complex flow field conditions within the turbine, thereby altering heat transfer dynamics. Consequently, the transient operating characteristics of the gas turbine are affected. Rapid changes in operating conditions further complicate the heat transfer processes, making dynamic simulations that account for heat transfer more challenging.

During the heat transfer process within the gas turbine flow path, the temperature of the working fluid fluctuates due to factors such as changes in compressor speed and the fuel supply rate to the combustion chamber [6]. The metal walls, with their higher heat capacity, are affected by thermal inertia. As a result, their temperature does not immediately respond to fluctuations in the working fluid temperature, leading to a delayed thermal response. This delay creates a noticeable temperature gradient between the working fluid and the walls, which triggers heat transfer effects. These effects not only cause a delayed response in turbine speed and the outlet temperature of the rotor components, but also alter the enthalpy of the working fluid, thereby affecting its work capacity and shifting the operational characteristics of the rotor components [7,8]. The direction of heat transfer induced by thermal inertia depends on whether the transition is accelerating or decelerating. During acceleration from steady-state operation, thermal equilibrium is maintained between the working fluid and the metal walls. However, as the turbine accelerates, this equilibrium is disrupted, and the temperature of the working fluid exceeds that of the metal walls. This creates a temperature difference that drives heat transfer from the hotter working fluid to the cooler walls until thermal equilibrium is restored. Conversely, during deceleration, the direction of heat transfer reverses [9]. Since heat transfer occurs within the gas turbine flow path, full-system tests can only measure changes at the turbine inlet and outlet, making it impossible to precisely capture variations in the flow and temperature fields within the path [10,11]. Therefore, further investigation into the mechanisms by which thermal inertia influences rotor component characteristics is essential.

The intensity of heat transfer varies across different components of a gas turbine due to differences in gas temperature, flow field conditions, and flow path geometry. In the compressor, the gas is drawn directly from external air, resulting in a relatively low temperature. Consequently, the heat transfer effect between the gas and metal components is relatively weak, being much less intense than that in the combustion chamber or turbine. However, as the air passes through the compressor and performs work, its temperature and pressure undergo significant changes, which influence the heat transfer between the compressor and the working fluid, thereby affecting turbine performance [12,13,14]. During transient processes, the gas in the compressor’s main flow path heats up due to the work performed by the compressor blades. Due to thermal inertia, the temperature of the metal components lags behind that of the main airflow, creating a temperature difference that drives heat transfer [15]. Maccallum was the first to investigate how transient heat transfer between the working fluid and metal components during the acceleration and deceleration of a dual-shaft gas turbine shifts the surge boundary towards the operating line [16]. Grant later examined the impact of transient heat transfer on the compressor’s characteristics, with a focus on changes in the blade boundary layer structure [17]. The findings of Maccallum and Grant regarding the surge line shift caused by transient heat transfer were experimentally evaluated [18]. By analyzing multiple “Bodie” transient processes, they estimated the heat transfer based on the average wall temperature of the compressor and aerodynamic parameters under steady-state conditions. Although their results did not establish a statistical correlation between transient heat transfer and surge line shift, they observed that, during compressor surging, the time-averaged heat transfer quantity was greater. In contrast, Shah evaluated steady-state heat transfer and the variation in blade inlet attack angles using a more accurate blade boundary layer model but did not account for the effect of transient heat transfer on compressor instability [19]. After combustion and mixing in the combustion chamber, the working fluid exits with uneven temperature and flow field characteristics, resulting in a gas flow containing localized high-temperature cores, hot spots, and vortex flows with increased turbulence [20]. As the hot spots enter the turbine rotor blade passage, the high-temperature thermal flow separates from the main gas stream due to the rapid rotation of the rotor blades, creating a distinct migration path. Additionally, centrifugal forces cause the hot spots to concentrate on the lower side of the rotor blade pressure surface, forming a thermally driven secondary flow [21,22,23]. This secondary flow, influenced by thermal effects, interacts with the secondary flow generated by the overall fluid movement, altering the thermal distribution on both the blade surface and within the blade passage, ultimately reducing turbine stage efficiency. When the vortex flow reaches the leading edge of the high-pressure turbine stator blades, the airflow decelerates, creating a static pressure gradient along the blade height. This results in vortex flow with secondary flow characteristics on both the pressure and suction surfaces. The static pressure gradient along the blade height is further affected by shifts in the stagnation line at the leading edge [24].

Visser et al. used a simplified heat transfer model for a twin-shaft gas turbine to investigate the heat transfer effects of various components during a typical acceleration process and fuel schedule [25]. The low-pressure compressor, with the lowest temperature, experiences the lowest heat transfer coefficient, resulting in minimal heat flux density. Early gas turbine simulation models typically overlooked heat transfer effects (e.g., using adiabatic models) or simplified conditions for computational convenience. These traditional models are based on several assumptions: conduction heat transfer is considered only radially; no conduction occurs between components; no heat is lost to the environment; the temperature distribution of all components in the main gas path is linear; the mass of all components is linearly distributed along the axial direction; and there is no heat loss in the air or cooling flow system. Such simplifications fail to accurately capture the increasingly complex flow fields and heat transfer effects within the flow path, as well as their influence on gas turbine operating conditions. To effectively assess gas turbine performance and stability during the conceptual and preliminary design phases, more sophisticated simulations are required. Schwab [26] was one of the pioneers in studying the heat transfer effects in gas turbines, emphasizing their importance in transient performance modeling. Additionally, dynamic simulations of gas turbines with regenerative heat, providing differential equations for turbine components and methods for calculating cooling gas flow, turbine blade temperatures, and regenerator performance, have also been conducted [27,28,29,30].

To accurately characterize the response of a marine gas turbine under transitional operating conditions, more detailed simulations are required to closely replicate the physical processes governing the turbine’s internal flow field and heat transfer effects, while also accounting for the complex heat transfer conditions within the flow path. This study primarily focuses on the dynamic simulation of a marine gas turbine under transitional operating conditions, using an actual gas turbine as the research object. It examines the impact of thermal inertia on turbine performance across various transitional conditions, considering both the mode and extent of its effects. In the three-dimensional simulation model of the gas turbine flow path, specific wall surfaces are designated as non-adiabatic, with the direction and magnitude of the temperature difference or heat flux density determined being based on experimental and computational data. As the working fluid flows over these walls, heat transfer occurs due to the predetermined temperature difference or heat flux density, resulting in a change in the entropy of the fluid. This change, in turn, alters the aerodynamic characteristics of the components relative to an adiabatic model. In the computational fluid dynamics simulation, heat transfer between the working fluid and the wall surface due to thermal inertia is modeled, and its impact on the dynamic characteristics of the impeller components is analyzed. A three-dimensional finite element simulation method is employed, focusing on the impeller components most susceptible to temperature difference-driven heat transfer. By incorporating various heat transfer conditions within the flow path, a three-dimensional simulation model of the impeller components reflecting the effects of thermal inertia is developed. The flow and temperature fields throughout the entire flow path are obtained by discretizing the governing control equations.

## 2. Numerical Computation Method

### 2.1. Turbulence Model Selection

The impeller components exhibit a complex flow field due to factors such as high-speed working fluid, intricate geometry, and impeller rotation, leading to fully turbulent flow [31]. This flow pattern induces frequent exchanges of momentum and energy between the fluid and the surrounding media, resulting in small-scale, high-frequency fluctuations [32,33,34]. The objective of this study is to examine the effect of heat transfer, driven by thermal inertia, between the wall surface and the working fluid on component performance. The focus is placed on the flow field near the wall boundary, while other regions of the flow field are subjected to lower accuracy requirements. To optimize the balance between computational effort and result accuracy, a two-equation turbulence transport model is employed.

The impeller component consists of two parts, the gas compressor and the turbine, each with distinct internal flow field characteristics. In the compressor, the working fluid temperature is lower, and the heat transfer between the fluid and the wall is less intense compared to that between the fluid and the turbine. Additionally, the flow path is longer, passing through multiple stages of rotors, and the flow field is significantly influenced by the Coriolis force [35,36,37]. Therefore, the compressor component model is best represented by the k-ε model with swirl correction, which effectively calculates rotational shear flow and boundary layers.

In the turbine component, the working fluid temperature is higher, resulting in more intense heat transfer between the fluid and the wall. The overall flow path is shorter, typically comprising one or two stages, and the flow field is less influenced by Coriolis forces compared to the compressor. However, due to the higher fluid velocity, flow separation near the wall is more likely to occur. Therefore, the turbine component is best modeled using the SST k-ω model, which effectively captures near-wall flow separation [38]. The SST k-ω model is a hybrid approach that employs the standard k-ω model near the wall to accurately simulate flow separation, while using the standard k-ε model further from the wall, thus achieving greater accuracy without significantly increasing computational effort [39]. This turbulence model setting is based on the prior experience and research findings of our team [26,27,28,30,36]. This numerical simulation approach has also been experimentally validated.

At present, the numerical calculations regarding the performance of the compressor are relatively accurate. However, the prediction accuracy of the SST k-ω model in real high-temperature and high-speed turbine environments still requires validation through experimental data. The current study is limited by experimental conditions, and validation experiments have not yet been conducted. In the future, to enhance the credibility of the turbine simulation model, a layered validation strategy will be further adopted. First, the SST k-ω model’s ability to predict surface pressure distribution will be validated based on classic blade-row cases. Secondly, the near-wall separation and heat transfer mechanisms will be validated through a low-temperature and low-speed test system (by obtaining flow field PIV data and wall temperature measurement results).

Finally, the temperature field distribution of the blade will be compared using thermal infrared imager temperature measurement technology in short-duration high-temperature tests.

### 2.2. Setting of Boundary Conditions of Adiabatic Wall

**Figure 1:**
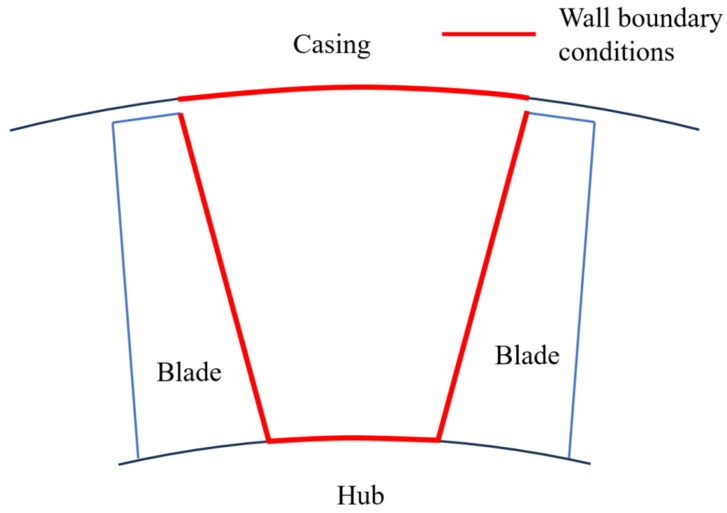
Schematic diagram of metal wall where thermal boundary conditions are set. (The black lines represent the casing and the blue lines represent the blades).

In the flow path of the impeller component, all wall surfaces in direct contact with the working fluid and subject to convective heat transfer must be assigned thermal boundary conditions. As shown in Figure 1, these surfaces primarily include the side surfaces of the blades, certain sections of the casing between the blades, and the surface of the hub. Additionally, due to gaps between the blade rows, the metal wall surfaces contacting the working fluid are limited to the casing and hub. Consequently, thermal boundary conditions must be applied to the inner walls of the casing and hub between the blade rows. At the tip clearance, the working fluid also encounters the tip clearance and part of the casing wall, undergoing convective heat transfer. However, inside the compressor, the air temperature in the flow path is not high. The contact region between the tip clearance and the casing wall is also relatively small. The leakage flow at the tip clearance moves from the pressure side to the suction side, and the working fluid temperature is close to the low inlet temperature, with a small temperature difference from the metal wall (which is typically heated by friction or influenced by downstream effects).

Additionally, this investigation is applicable to subsonic compressors with normal tip clearance design and standard temperature intake conditions. The leakage flow itself only accounts for 1–2% of the mainstream flow. And the flow path is short, resulting in a very brief heat exchange time. The heat transfer between the working fluid and the wall in the tip clearance region accounts for only 0.021% of the total enthalpy at the inlet. And changes in the tip clearance height do not significantly affect the distribution of the heat transfer coefficient in this region. Therefore, the heat transfer effect between the compressor and the metal components is weak and can be considered negligible.

### 2.3. Boundary Conditions of Coupled Heat Transfer Walls

For coupled heat transfer wall boundary conditions, temperature difference boundary conditions are employed. This approach uses the wall temperature, measured under the rated operating conditions of the gas turbine, as the initial boundary condition. By adjusting the temperature difference, various levels of temperature disparity between the wall and the working fluid are created. This method enables the examination of the deviation in the characteristic curve of the rotor component, accounting for or neglecting thermal inertia [40]. Furthermore, once the temperature difference boundary conditions are set, the total heat transfer between the working fluid and wall, along with the difference in the working fluid’s enthalpy, can be calculated. This allows for the determination of the proportion of thermal power change due to thermal inertia in the overall heat transfer variation, reflecting the impact of thermal inertia on the work capacity of working fluid [41,42,43]. However, this method has limited boundary condition variations for components that are not highly sensitive to temperature differences. Additionally, setting the temperature difference too high may cause the simulation to fail to converge. The small range of boundary condition variations makes it difficult to capture the shift in component characteristic curves due to changes [44].

Another method for setting thermal boundary conditions is the heat flux density approach. Based on aerodynamic boundary conditions, this method assigns a constant heat flux density to all metal surfaces surrounding the flow path that may exchange heat with the working fluid. Unlike the constant temperature difference method, the heat flux density method only defines a specific heat flux density without restricting the temperature state between the working fluid and the wall. This flexibility allows for a broader range of boundary condition variations while ensuring the convergence of simulation results, leading to more accurate and distinct shifts in the characteristic curve. However, because the total heat transfer between the working fluid and the wall is externally defined, the heat flux density method is less precise than the temperature difference method in capturing the relationship between total heat transfer and the enthalpy change in the working fluid. Moreover, directly measuring the heat flux density within the rotor component is challenging and typically requires the simulation results from the temperature difference method to be combined.

This study combines the temperature difference method and the heat flux density method to define the wall thermal boundary conditions. Initially, the temperature difference method is employed to set varying temperature differences between the working fluid and the wall, starting from the cold state, until the simulation fails to converge. Based on the simulation results from these temperature difference boundary conditions, the upper and lower limits of the heat transfer proportion between the wall and the working fluid relative to the total inlet enthalpy are calculated. Then, the total heat transfer range along the entire flow path is determined using the outlet total pressure, the Mach number, and the calculated limits of the heat transfer proportion. Finally, the heat flux density range is defined by computing the total surface area of non-adiabatic walls. To quantify this range, a heat flux density coefficient (q*) is introduced, which is expressed as follows:

(1) $$q^{*}=\frac{Q}{mh_{t,in}}$$

where q* represents the heat flux density coefficient. *h_t_*_,*in*_ denotes the total specific enthalpy of the working fluid at the inlet. *Q* is the total heat transfer rate. And *m* is the mass flow rate of the working fluid. The coefficient q* indicates the ratio of total heat transfer along the flow path to the total inlet enthalpy of the working fluid per unit time.

## 3. Establishment of 3D CFD Simulation Model for Impeller Parts

### 3.1. Three-Dimensional CFD Simulation Model of Compressor

**Figure 2:**
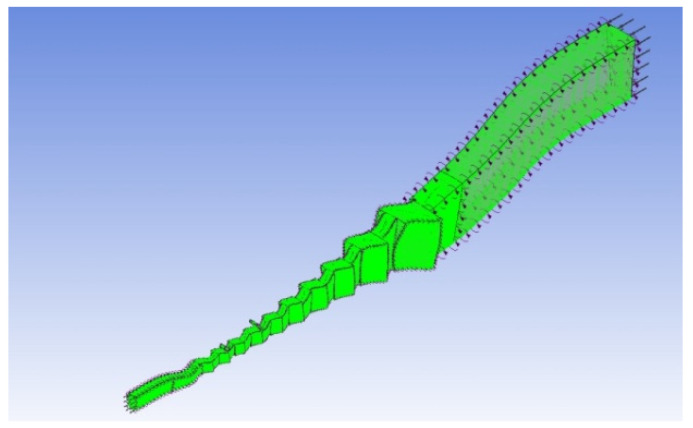
Overall compressor grid model.

The gas turbine primarily consists of a ten-stage compressor, a two-stage power turbine, and a single-stage high-pressure turbine. The compressor components are selected as the simulation target, and a one-pass three-dimensional simulation model is developed. The compressor includes the first-stage guide IGV blades, a ten-ring rotor, and a ten-ring stator arranged in a staggered pattern. A short inlet passage is positioned at the front end of the IGV blades, while a short diffuser passage is located at the trailing edge of the last-stage stator. In addition to the main flow path, the compressor features annular bleed flow paths at the outlet diameter of the sixth-stage stator (S5) and the eighth-stage stator (S7). As illustrated in Figure 2, the model employs a hexahedral structured grid with a total of approximately 3.5 million grids. The wall surfaces requiring thermal boundary conditions—such as specific sections of the casing, hub surface, and blade side surfaces—undergo boundary layer refinement.

The compressor grid setup includes interface settings for gas mixing and rotational domain configurations. Given the relatively long flow path and high pressure ratio of the compressor, a k-ε model with swirl correction is employed. Additionally, the interface model for each domain is set as Stage (Mixing-Plane). The convective terms of both the energy equation and the turbulence transport equation are solved using the high-resolution scheme. A pseudo-time step size of 1^−4^ s is applied. And when the mean squared residual of iterations is less than 1^−6^, the calculation is convergent.

The grid independence of the compressor mesh is verified using the SIMPLE algorithm. Initially, the velocity field at the outlet of the compressor flow path is calculated. The initial grid count is set to 940,000, and the grid count is progressively increased according to the pattern outlined in Table 1. The variation in outlet velocity is monitored as the grid count increases, while all other conditions remain constant. When the grid count exceeds 3.5 million, the simulation results stabilize, showing no further variation with additional grid refinement. Therefore, a grid count of 3.5 million is used in the model.

The inlet and outlet boundary conditions for the single-flow path compressor based on experimental data are given in Table 2. The outlet boundary conditions are set by modifying the outlet static pressure, enabling convergence for different cases. And the adiabatic wall boundary conditions are used as the baseline. Given the relatively long flow path and high inlet and outlet pressure ratios, the compressor design also includes annular bleed air passages at the outlet diameter of the sixth- and eighth-stage stators. The outlet mass flow rates for these two bleed air paths are set to 0.015 kg/s and 0.011 kg/s, respectively. Since these bleed air paths are separate from the main flow path, no wall heat boundary conditions are applied. The intake angle is determined using CFX’s built-in interpolation method and is expressed in vector form within the Cartesian coordinate system. Additionally, interpolation is used to model the relationship between gas viscosity and specific heat as they vary with temperature.

### 3.2. Three-Dimensional CFD Simulation Model of High-Pressure Turbines

**Figure 3:**
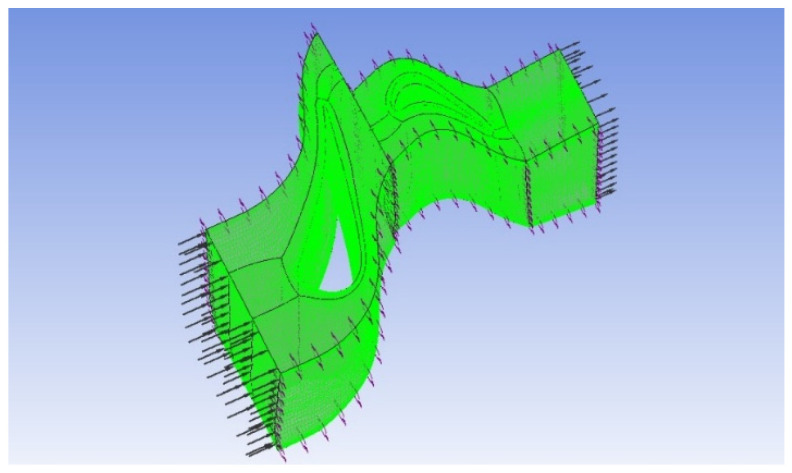
Overall mesh model of high-pressure turbines.

A three-dimensional simulation model was developed for the single-stage high-pressure turbine component of a specific gas turbine, focusing on a single-flow path. All flux-related parameters (such as mass flow rate, volumetric flow rate, and heat flux) are scaled by the number of blades. As displayed in Figure 3, to ensure simulation accuracy, a structured hexahedral mesh grid model, with approximately 860,000 grid points, is used. The minimum orthogonal angle is 22.30°, the maximum expansion ratio is 2.5, and the maximum grid aspect ratio is approximately 1072, with particular emphasis on refining the boundary layer near the wall surface.

The stator region is fixed, while the rotor region rotates at the rated speed, reflecting the distinct characteristics of each region. The interface between the rotor and stator utilizes the “Conservative Coupling by Pitchwise Row” method for data transfer between adjacent blade rows. Due to the imperfect matching of the inlet and outlet areas formed by each blade row, gaps exist, which necessitate a consideration of fluid mixing. The stage interface model is employed to address this. Given the high inlet working fluid velocity and temperature of the high-pressure turbine, and considering that the power turbine is a single-stage design with a short flow path, the inlet and outlet flow fields can significantly influence the internal flow field of the blades, potentially leading to leakage vortices at the outlet. To minimize this interference, the inlet transition section is set to 30% of the blade chord length, and the outlet transition section is set to 50% of the blade chord length.

In the solver settings, the energy equation’s convective term is discretized using the high-resolution scheme, while the convective term of the turbulence transport equation is approximated with a first-order upwind scheme. The convergence criterion is set such that the iterative root-mean-square residual is below 1^−6^. The grid independence of the high-pressure turbine is verified using the SIMPLE algorithm. The velocity field at the outlet of the high-pressure turbine is calculated, with an initial grid size of 215,000. The grid size is progressively increased, as shown in Table 3. And the simulation results are monitored to assess whether the outlet velocity changes with grid size under the same conditions. The results indicate that when the grid count exceeds 860,000, the simulation results stabilize, and further grid refinement does not affect the outcome. Therefore, a grid size of 860,000 is used in the model.

The inlet and outlet boundary conditions of the single-flow passage are set based on actual experimental data. The provided power turbine test data, derived from generalized parameters, yields initial conditions such as equivalent temperature and rotational speed. The experiments cover four distinct operating conditions, as detailed in Table 4.

### 3.3. Three-Dimensional CFD Simulation Model of Power Turbine

**Figure 4:**
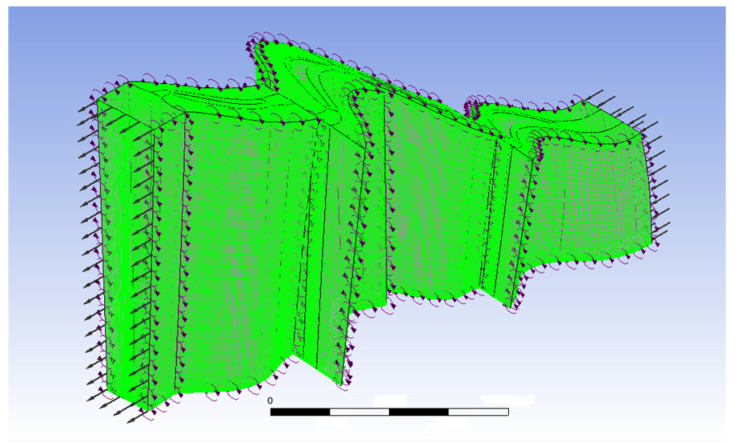
Overall grid model of two-stage power turbines.

A three-dimensional single-flow passage simulation model is developed using the power turbine component. The model comprises two rotor rings and two stator rings, arranged alternately. Due to variations in the radius of each stage’s rotor and stator, as well as the geometric characteristics of the blades, the number of blades differs across the four-stage turbine. Consequently, in the CFD simulation of the single-flow passage, the flux parameters at each stage (such as mass flow, volumetric flow, and heat flux) are multiplied by the corresponding number of blades. To ensure simulation accuracy, a structured hexahedral grid is used, comprising approximately 1.2 million grids. The minimum orthogonal angle is 22.30°, the maximum stretching ratio is 2.6, and the maximum grid aspect ratio is approximately 1284. The grid near the wall boundary layer is shown in Figure 4.

**Figure 5:**
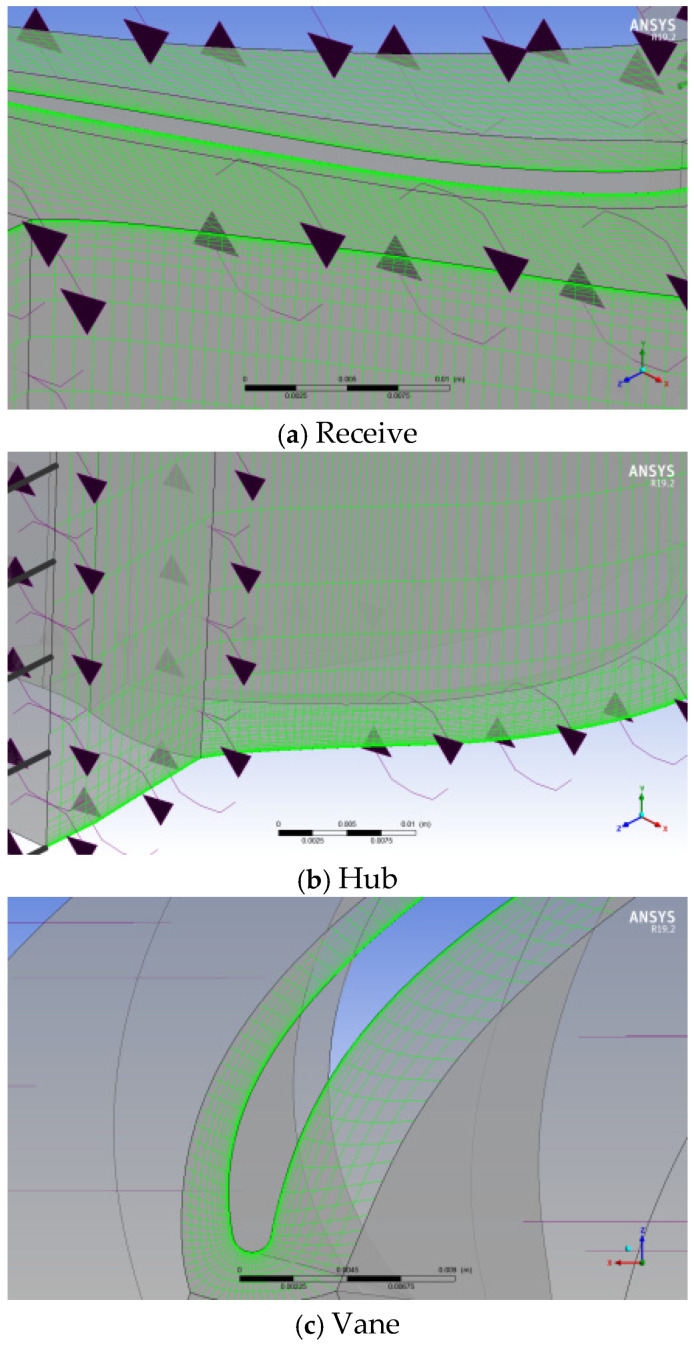
Wall boundary layer mesh encryption.

The metallic surfaces, such as the casing, hub, and blades, are in direct contact with the flow medium. The wall surface is strongly influenced by the boundary layer effect, making it highly sensitive to velocity and temperature gradients. To ensure accurate heat transfer calculations between the wall and the flow medium, the grid density in the normal direction of the wall is increased significantly. Consequently, a prism layer is generated during meshing, with the first grid layer positioned 1 × 10^−6^ m from the blade surface, as shown in Figure 5. The increased grid density in the wall boundary layer effectively captures the variations in the temperature and velocity of the flow medium in the normal direction of the wall.

The stator region is defined as a fixed state, while the rotor region is defined as a rotating state, with the rotational speed set to the rated value. The left and right interfaces of each region are set to periodic conditions, with the circumferential array arranged along the Z-axis according to the number of blades. The interface between the rotor and stator uses the Conservative Coupling by Pitchwise Row method to transfer data between adjacent blade rows. Furthermore, due to misalignment and gaps between the inlet and outlet of each blade row, fluid mixing is considered, and the stage (mixing–plane) interface model is applied. To prevent interference between the flow field at the inlet and outlet and the internal flow of the blade rows, a transition region corresponding to 20% of the moving blade chord length is defined at both the inlet and outlet of the component.

The convection term of the energy equation is solved using the high-resolution scheme, while the convection term of the turbulence transport equation is discretized with a first-order upwind scheme. The solver employs a variable step-size pseudo-time stepping method, with the convergence criterion defined as the mean square residual of the iterations being less than 1^−6^. During the simulation, the momentum and energy residuals in all three directions begin to oscillate after approximately 150 steps. The momentum residual converges to 1^−4^, and the energy residual approaches 1^−6^.

The grid independence of the power turbine mesh is verified using the SIMPLE algorithm. Based on computational performance, the initial grid count is set to 426,000, and the grid count is progressively increased according to the pattern shown in Table 5. With all other parameters held constant, the simulation results are analyzed to determine whether the outlet velocity changes with variations in the grid count. The results indicate that when the grid count exceeds 1.2 million, the simulation results remain stable, with no further changes observed. Therefore, a mesh count of 1.2 million is adopted for the model.

The inlet and outlet boundary conditions for the single-channel turbine are set based on actual experimental data. The provided power turbine test data are derived from generalized parameters to calculate initial conditions, such as equivalent temperature and rotational speed, for four operating conditions, as shown in Table 6. The outlet boundary conditions are established by gradually adjusting the static pressure at the outlet, ensuring that the expansion ratio during the convergence process approximates the experimental results. This approach ensures that the simulation conditions for the power turbine align with the experimental conditions across all four operating states. Additionally, to match the inlet conditions with the guide vanes at the turbine inlet in the experiment, the inlet boundary conditions incorporate varying intake angles with respect to blade height. Using the interpolation method provided by CFX, a three-dimensional distribution of the intake angle is obtained and represented as vectors in a Cartesian coordinate system. Similarly, interpolation methods are applied to determine the relationship between gas viscosity, specific heat, and temperature variations.

## 4. Results and Analysis

### 4.1. Technical Work and Heat Flux Coefficient with Temperature Difference Boundary Condition

**Figure 6:**
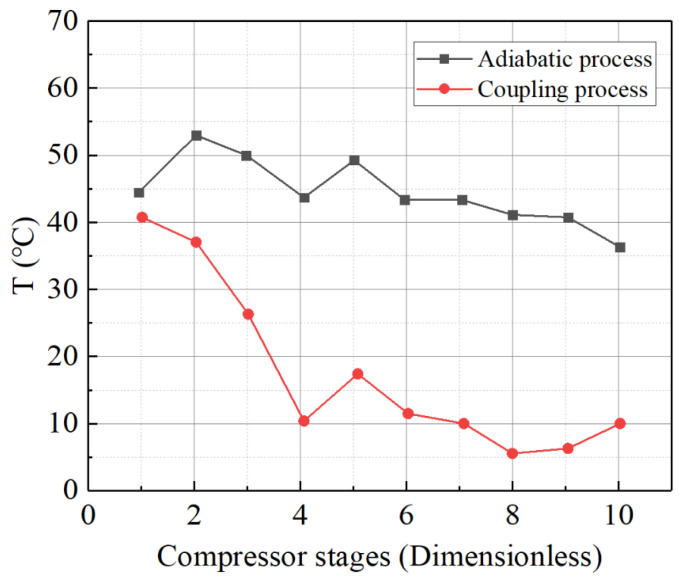
Variation trend of temperature difference between inlet and outlet of compressor at all levels with stage (cold state).

An interpolation method is employed to incorporate the variation in specific heat with temperature into the boundary conditions for the total temperature at each stage inlet. For instance, in the case of a thermal boundary condition with a constant wall temperature (set at 30 °C in the cold state), simulation calculations were conducted to determine the temperature differences in the working fluid at the inlets and outlets of each stage, as shown in Figure 6. The results demonstrate that, in the adiabatic model, the temperature differences at the inlets and outlets of each stage gradually decrease, exhibiting a relatively uniform linear distribution across the compressor flow path. In the non-adiabatic model, the temperature differences at the inlets and outlets of each stage are generally lower, showing a similar decreasing trend and maintaining a relatively flat linear distribution, except for the fourth stage. These results indicate that the temperature difference in the working fluid in the compressor flow path follows a nearly linear distribution, making the method of setting specific temperature difference or heat transfer rate for the wall reasonably reliable.

Four types of thermal wall boundary conditions were applied: adiabatic, non-adiabatic, hot-state, and cold-state conditions. However, during the simulation, it was observed that when the wall temperature difference exceeds 150 K, the results under various back pressure conditions fail to converge. The initial analysis suggests that the compressor is sensitive to high-temperature fixed wall conditions. Unlike turbines, the compressor does not experience significant temperature fluctuations between the wall and the working fluid in the flow path during practical operation, due to the absence of fuel a supply. Consequently, thermal wall boundary conditions with a temperature difference of Δ ≥ 150 K may not accurately represent the compressor’s actual operating conditions.

**Figure 7:**
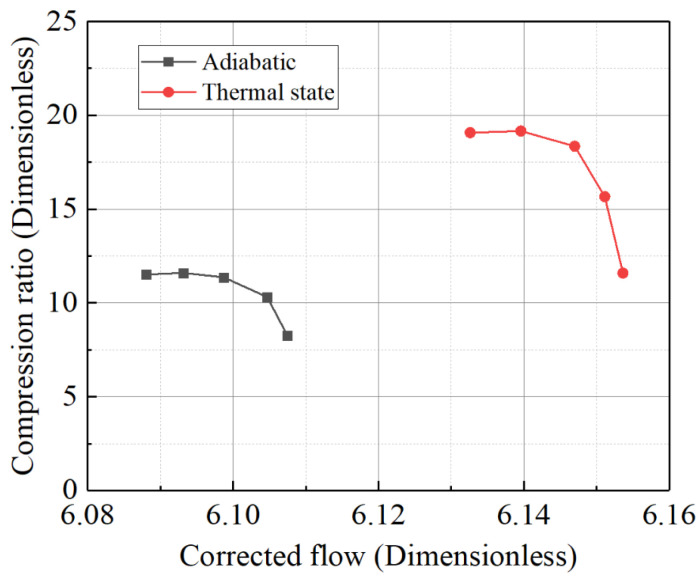
Compressor compression ratio characteristic curve at 1.0 speed.

In conducting CFD simulations of the compressor using the constant temperature difference method, various wall thermal boundary conditions were considered. All simulations were conducted at a rated speed, and the adiabatic, non-adiabatic, and hot/cold-state conditions were analyzed together. The simulation results are shown in Figure 7. Notably, when the compressor outlet pressure exceeds 1.93 MPa, a choking phenomenon occurs, and the flow rate becomes independent of the outlet pressure. Under the cold-state boundary conditions, the outlet pressure consistently exceeds 1.93 MPa, meaning the characteristic curve under these conditions shows only one choking point.

The simulation results indicate that, under certain operating conditions, the temperature distribution of the casing and hub is higher compared to that under the conditions of the simulation speed, placing these components in a state where their temperature exceeds that of the working fluid in the flow path. As a result, heat transfer occurs, with the working fluid absorbing heat from the wall. In the other two heat absorption scenarios, the wall temperature increases by 100 °C overall. As shown in the figure, during the transition phase where heat is released from the wall to the working fluid, both the compression ratio and efficiency curves shift to the left. Conversely, when the wall absorbs heat from the working fluid, the curves shift to the right. Considering the compressor’s characteristics, it can be inferred that heat release from the wall causes the gas temperature to rise continuously during compression, leading to a reduction in gas density inside the compressor. Although the volumetric flow rate remains constant, the mass flow rate decreases, making compression more difficult. This results in reduced compression efficiency and a lower compression ratio. On the other hand, when the wall absorbs heat from the gas, the process can be understood as a cooling effect during compression. As the compression process shifts from an adiabatic isentropic line to an isothermal line, the compressor’s power consumption is reduced compared to that in the isentropic process, improving compression efficiency.

The total enthalpy change in the compressor components is provided in Table 7. In the design of compressors and thermal management systems, it is crucial to have a deep understanding of the thermodynamic nature of wall heat transfer and its relationship with system performance and long-term operational reliability. The thermodynamic impact of wall heat transfer modes (heat release and heat absorption) on compressor performance primarily manifests in alteration in entropy generation and irreversible losses during the compression process.

Wall heat release (heating the working fluid) significantly increases entropy, mainly by intensifying irreversible processes such as viscous dissipation, flow separation, and leakage, leading to a decrease in efficiency and an increase in outlet temperature. This has profound negative effects on the system, severely threatening the lifespan of thermal components, worsening the working environment of bearings, increasing the risk of thermal stress fatigue, and potentially reducing operational stability (surge margin), ultimately leading to higher maintenance costs and a shortened overall operational lifespan. On the other hand, wall heat absorption (cooling the working fluid) helps to reduce entropy by mitigating the irreversible losses, improving efficiency, and lowering outlet temperature. The main system benefits include significantly extending the lifespan of thermal components, improving bearing reliability, and potentially enhancing stability.

The working principle of a compressor differs from that of a turbine in that it performs work on the working medium through cascade rotation, resulting in positive technical work that is greater than the total enthalpy change. However, in the compressor components, the working medium temperature is relatively low, with a small temperature difference between the medium’s and the wall. Consequently, the heat transfer is less intense than that in the turbine components, and the overall heat exchange accounts for only about 0.1% of the total enthalpy of the incoming fluid. Therefore, when defining the wall thermal boundary conditions for compressor components, the constant heat flux method is employed, with the value of q* typically falling in the range ±0.001.

**Figure 8:**
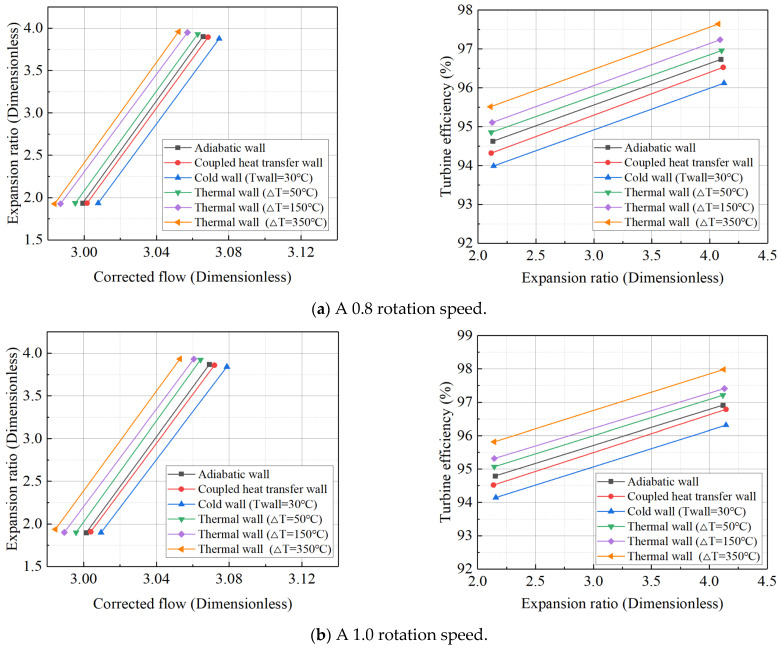
Expansion ratio characteristic lines and efficiency characteristic lines of high-pressure turbines at reduced speeds of 0.8 and 1.0 under different boundary conditions.

For the high-pressure turbine, the six temperature difference settings used for the wall heat boundary condition, employing the constant temperature difference method, are the same as those for the power turbine. The data for the high-pressure turbine’s adiabatic/steady-state boundary conditions under various operating conditions, along with the turbine expansion ratio and efficiency characteristics at corrected rotational speeds of 0.8 and 1.0, are presented in Table 8. As shown in Figure 8, the high-pressure turbine’s characteristics under different thermal wall boundary conditions reveal that the deviation pattern is similar to that of the power turbine. However, the high-pressure turbine is less sensitive to large temperature differences compared to the power turbine. For the power turbine, the simulation diverges at high rotational speeds when the temperature difference reaches 350 K, while the high-pressure turbine model continues to show good convergence at this temperature difference. Nevertheless, if the temperature difference is further increased, the simulation of the high-pressure turbine also diverges. Therefore, the upper limit of the temperature difference for the high-pressure turbine is set at 350 K.

**Figure 9:**
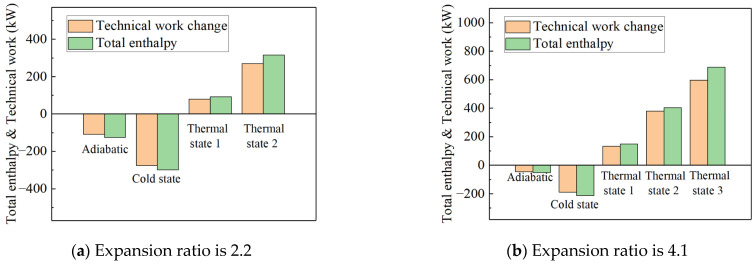
Proportion of technical work change in high-pressure turbines to total enthalpy change under different boundary conditions.

Figure 9 illustrates the proportion of the change in technical work for the high-pressure turbine relative to the total enthalpy change. The overall enthalpy change in the working fluid in the high-pressure turbine flow path is relatively small, resulting in a slightly lower proportion of technical work change compared to the total enthalpy change. This is primarily due to the high-pressure turbine having only one stage, i.e., fewer stages, and a shorter flow path, which reduces the contact area between the working fluid and the wall. Based on the calculated heat transfer proportion relative to the total enthalpy at the inlet, when using the constant heat flux density method to set the wall heat boundary conditions for the high-pressure turbine, the range of q* values is approximately between −0.01 and −0.06.

The adiabatic wall boundary condition is the standard simulation setup used in industry, while the non-adiabatic wall boundary condition represents the stable heat transfer process between the flow path gas and the metal wall during steady gas turbine operation. Thermal boundary conditions model transient processes in which the flow path working fluid temperature rapidly decreases. Due to thermal inertia, the wall temperature forms a noticeable temperature difference with the working fluid and transfers heat to it. The cold-boundary condition, on the other hand, represents a transient process where the flow path working fluid temperature rapidly increases, while the wall temperature remains low, absorbing heat from the working fluid. The simulation results for the non-adiabatic steady-state boundary condition show less than a 3% difference compared to the adiabatic boundary condition, as shown in Table 8. This minor discrepancy further supports the validity of ignoring heat transfer effects in traditional steady-state simulations while still maintaining good accuracy.

**Figure 10:**
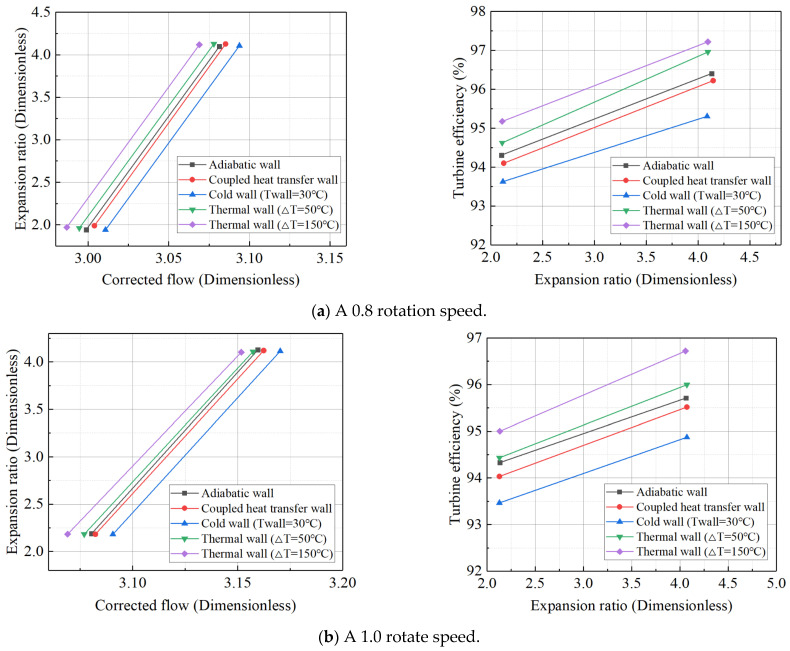
Expansion ratio characteristic lines and efficiency characteristic lines of power turbines at reduced speeds of 0.8 and 1.0 under different boundary conditions.

Due to the minimal difference between non-adiabatic and adiabatic conditions, cold and thermal wall boundary conditions were sequentially applied to better observe heat transfer between the flow path working fluid and the metal wall. Transient heat transfer was simulated under conditions such as the turbine’s cold start (where the metal temperature remains at ambient temperature) and deceleration (where the fuel flow and flow path working fluid temperature suddenly drop, but the metal temperature remains high). The simulation results, shown in Figure 10, reveal that when the temperature difference exceeds 350 K, the calculation diverges. This indicates that 350 K is the maximum temperature difference achievable when setting wall heat boundary conditions using the constant temperature difference method. Analysis of the power turbine’s characteristic curves under different thermal wall boundary conditions revealed the following: Compared to the adiabatic boundary condition, the characteristic curve shifts to the right (indicating an increase in equivalent flow rate) when the working fluid releases heat to the metal wall, and shifts to the left (indicating a decrease in equivalent flow rate) when the heat transfer direction is reversed. Similarly, the characteristic curve shifts downward (indicating a decrease in efficiency) when the working fluid releases heat to the metal wall, and shifts upward (indicating an increase in efficiency) when the heat transfer direction is reversed. The observed shift in equivalent flow rate may be attributed to changes in mass flow, caused by variations in the density of the flow path working fluid, as well as potential aerodynamic parameter changes that affect the actual flow velocity. Additionally, in the cold state, the relative velocity at the first-stage stator exit is higher.

**Figure 11:**
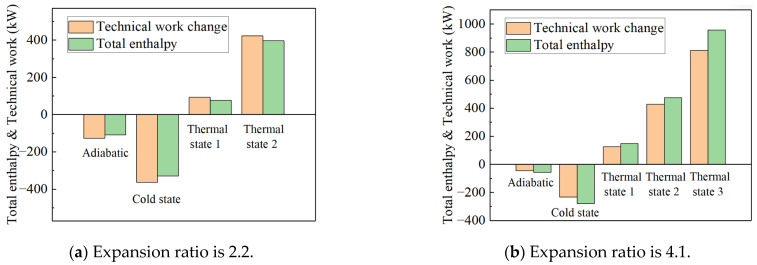
Ratio of technical work change to total enthalpy change of power turbines under different boundary conditions.

The impact of transient heat transfer on turbine efficiency can be explained through the concept of total enthalpy loss. When the working fluid in the flow path transfers heat to the metal wall, its total enthalpy decreases, resulting in a loss of power. Conversely, when the working fluid absorbs heat from the metal wall, its total enthalpy increases, leading to a power gain. However, according to the second law of thermodynamics, not all the heat absorbed by the working fluid from the wall can be converted into useful work. Regardless of whether the working fluid absorbs or releases heat, the total heat exchange manifests in two forms: (1) a change in the total enthalpy of the working fluid and (2) a change in the technical work output. To quantify the contributions of these two components to the total heat transfer, the adiabatic boundary condition was used as a baseline, and the deviations of various parameters were analyzed, as shown in Figure 11. For example, under an operating condition of a 1.0 rotational speed and a pressure ratio of 4.1, 943.098 kW of heat was transferred from the wall to the working fluid under Thermal State 3. Of this, 801.308 kW was converted into an increase in the total enthalpy of the working fluid, which was reflected in the temperature rise in the exhaust gas, while the remaining 140.67 kW was converted into technical work performed by the working fluid on the turbine. Notably, the latter value is nearly equal to the former, indicating that under these conditions, approximately 15% of the absorbed heat was converted into technical work by the working fluid.

The transient heat transfer (working fluid heat absorption/release) in turbines essentially involves the generation of entropy and irreversible changes. The heat transfer process itself, along with the changes in the working fluid state caused by heat absorption/release, such as the deviation from the isentropic process in the expansion process, mixing, and secondary flow losses, all contribute to entropy increase.

Wall heat absorption (cooling the blades) is a key method for extending the lifespan of high-temperature turbine blades. By significantly lowering the metal temperature, it can suppress creep, corrosion, and material degradation, significantly extend the overhaul interval and reduce maintenance frequency and costs. In contrast, wall heat release (heating the blades) shortens blade life, dramatically increases maintenance demands and costs, and may even endanger operational safety. In the heat transfer process, a large portion of energy is converted into enthalpy change, while most of the energy is not effectively converted into work, resulting in increased exhaust losses and significant entropy generation. Although the portion converted into work directly increases output, its conversion efficiency is limited. And the conversion process itself also generates irreversible losses. Therefore, the core issue in controlling turbine wall heat transfer lies in balancing the cost of blade cooling with the lifespan benefits it provides.

The simulation results indicate that the total enthalpy change increases as the temperature difference rises. Except for thermal boundary condition 3, which has the maximum temperature difference, the proportion of technical work variation is greatest under the cold boundary condition. This finding is consistent with the system test results presented in Section 2. The temperature difference in thermal boundary condition 3 is too large, causing the simulation to diverge at a pressure ratio of 2.2. This further underscores the limitations of the constant temperature difference method, which has a narrow range of applicability. Based on the heat transfer values, the proportion of heat transferred to the total enthalpy at the inlet for the power turbine component, using the constant heat flux density method to set the wall heat boundary condition, yields q* values in the range of approximately −0.01 to −0.09.

### 4.2. Calculating the Component Characteristic Line with Heat Flux Boundary Conditions

Both adiabatic and thermal simulation results, along with experimental tests, indicate that the Mach number of the main working medium in the compressor is less than 1. As a result, the thermal boundary conditions for the simulation can be determined based on the value of q*. The constant heat flux density method was employed to apply different thermal wall boundary conditions in the CFD simulations across various operating conditions. The corresponding wall heat flux density, Φ, was set according to the value of q* (see Table 9).

**Figure 12:**
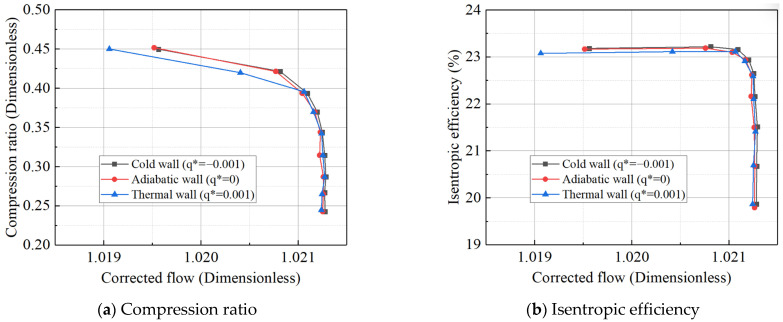
Compressor characteristic curve at 1.0 speed.

Based on the inlet Mach number and outlet total pressure during actual operation, the q* was calculated to be approximately on the order of 1 × 10^−3^. The wall thermal boundary conditions from Table 9 were sequentially applied to the simulation model, resulting in characteristic line shifts in the compressor under different q* values, as shown in Figure 12. The simulation results indicate that, during the transition of heat release from the wall to the working fluid, both the compression ratio and efficiency curves of the compressor shift to the left. In contrast, when the working fluid absorbs heat from the wall, the curves shift to the right, which aligns with the conclusions from the constant temperature difference method. Notably, as a rotor component sensitive to temperature differences, the compressor’s characteristic line shifts are more pronounced with the constant heat flux density method compared to the constant temperature difference method, which exhibits a broader range of values. Conversely, turbine components sensitive to temperature differences show the opposite trend. This suggests that, for thermal inertia simulation studies, the boundary condition setting method should be adjusted to account for the specific characteristics of the components.

Both the adiabatic and thermal state simulation results, along with experimental data, indicate that the Mach number of the mainstream working fluid inside the high-pressure turbine is less than 1. Therefore, the thermal boundary conditions for the simulation can be determined based on the value of q*. The constant heat flux method was applied to introduce varying thermal wall boundary conditions into the CFD simulations under different operating conditions. The corresponding wall heat flux density, Φ, was set according to the different values of q*, as outlined in Table 10.

**Figure 13:**
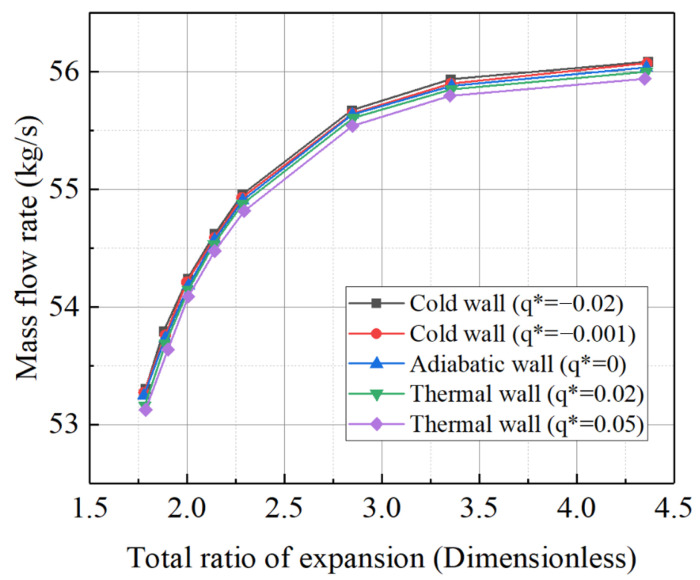
Characteristic curve of high-pressure turbines at 1.0 speed.

Based on the inlet Mach number and outlet total pressure during actual operation, the q* was calculated to be approximately 1 × 10^−2^. The wall heat boundary conditions from Table 10 were sequentially applied to the simulation model, resulting in shifts in the characteristic line of the power turbine at different q* values, as shown in Figure 13. The simulation results indicate that the direction shift of the high-pressure turbine characteristic line is similar to that of the power turbine. However, when compared to the adiabatic state, it was found that for every 0.01 increment in q*, the average shift in the high-pressure turbine characteristic line was only 60.55% of that observed for the power turbine. This explains why the high-pressure turbine is less prone to divergence than the power turbine under boundary conditions with larger temperature differences: the high-pressure turbine is less sensitive to thermal inertia. Moreover, the impact of thermal inertia on the gas turbine rotor components is influenced by the component volume and the contact area between the working fluid and the wall. Smaller components or reduced contact areas between the working fluid and the wall result in a diminished influence of thermal inertia on the components.

Both the adiabatic and thermal simulation results, along with experimental data, indicate that the Mach number of the mainstream working fluid inside the power turbine is less than 1. Therefore, the thermal boundary conditions for the simulation can be determined based on the value of q*. The constant heat flux method was employed to apply varying thermal wall boundary conditions in the CFD simulations under different operating conditions. Based on the value of q*, different wall heat flux densities, Φ, were set, as detailed in Table 11.

**Figure 14:**
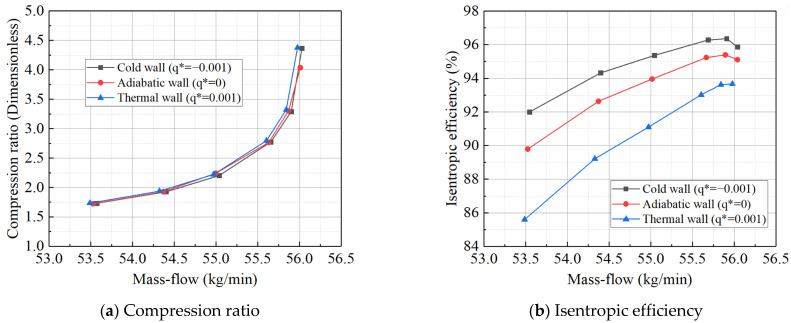
Characteristic curve of power turbine at 1.0 speed.

**Figure 15:**
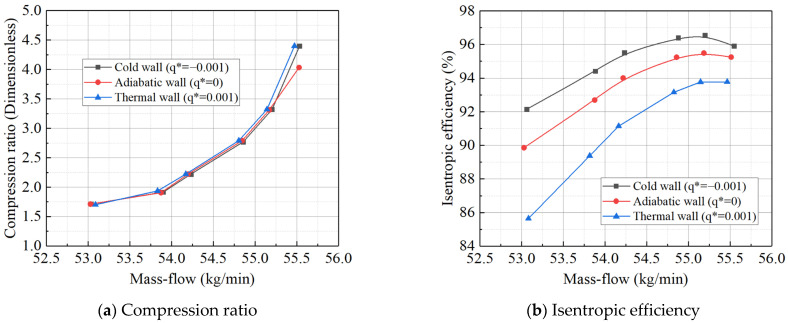
Characteristic curve of power turbine at 0.8 speed.

Based on the inlet Mach number and exit total pressure during actual operation, q* is calculated to be approximately 1 × 10^−2^. The wall heat boundary condition values from Table 4 are sequentially applied to the simulation model for computational analysis, resulting in characteristic line shifts in the turbine at different q* values, as shown in Figure 14 and Figure 15. Using the adiabatic boundary condition as a reference, when the working fluid releases heat to the metal wall, the flow–pressure ratio characteristic line shifts to the right (indicating an increase in flow). Conversely, when the working fluid absorbs heat, the flow–pressure ratio characteristic line shifts to the left (indicating a decrease in flow). Similarly, the flow–isentropic efficiency characteristic line shifts upward (indicating increased efficiency) when the fluid releases heat to the metal wall, and downward (indicating decreased efficiency) when the fluid absorbs heat. These results are consistent with those obtained using the constant temperature difference method, confirming the accuracy of the simulation. According to the simulation results, the direction of the turbine characteristic line shift is determined by the heat transfer direction. While the impact of different rotational speeds on the characteristic line shift is minimal under the same heat transfer conditions, some differences remain. Specifically, compared to the adiabatic state, the average pressure ratio and isentropic efficiency characteristic line shifts at a speed of 1.0 are 12.1% and 4.5% smaller, respectively, than those when the speed is 1.1. Furthermore, the absolute value of the heat flux density set for heat release from the wall (corresponding to the deceleration process during actual machine operation) is twice that for heat absorption from the wall (corresponding to the acceleration or start-up process). However, its effect on the characteristic line shift is only 163% of that when heat is absorbed by the wall.

## 5. Conclusions

The thermal inertia of gas turbines was investigated, and its impact under various transitional operating conditions was analyzed through three-dimensional simulations. A dynamic simulation model incorporating thermal inertia was developed, enabling an accurate prediction of the response delay time and the variation in various parameters affected by thermal inertia. The following conclusions were drawn:

In the adiabatic model, the temperature difference between the inlet and outlet at each stage gradually decreases, with a relatively uniform trend across the entire compressor flow path, displaying a nearly linear distribution. In the coupled heat transfer model, the temperature differences at each stage are generally lower and decrease gradually, following a mild linear trend. Even for the compressor, a component with many stages and relatively high pressure, the approach of setting all wall surfaces to the same temperature difference or heat transfer rate proves to be relatively reliable. The direction of the compressor characteristic line shift is determined by the heat transfer direction. When using adiabatic boundary conditions as a reference, if the compressor releases heat from the wall to the working fluid, both the compression ratio and efficiency curve shift to the left. Conversely, if heat is absorbed from the working fluid by the wall, the curves shift to the right. In simulations of thermal inertia for compressor components, although the boundary conditions based on the constant temperature difference method cover a narrower range, the shift in the characteristic lines is more pronounced compared to that using the constant heat flux density method.

For the power turbine, the direction of the characteristic line shift also depends on the heat transfer direction. Using adiabatic boundary conditions as a reference, the flow-to-pressure ratio characteristic line shifts to the right (indicating increased flow) when heat is released from the working fluid to the metal wall, and shifts to the left (indicating decreased flow) when heat is absorbed. Similarly, the flow-to-isentropic efficiency characteristic line shifts upward (indicating increased efficiency) when heat is released to the metal wall, and shifts downward (indicating decreased efficiency) when heat is absorbed.

Under the same heat transfer conditions, different rotational speeds have a minor effect on the degree of characteristic line shift. Compared to the adiabatic state, at a rotational speed of 1.0, the average shifts in the pressure ratio and isentropic efficiency characteristic lines are 12.1% and 4.5% smaller, respectively, than those at a speed of 1.1. When the wall releases heat (corresponding to the deceleration process in actual engine operation), the absolute value of the heat flux density is twice that observed when the wall absorbs heat (corresponding to the acceleration or start-up process). However, the impact on the characteristic line shift is only 163% of that observed when the wall absorbs heat. When the working fluid exchanges heat with the wall, approximately 6–15% of the exchanged heat is converted into the acquisition or loss of technical work. This proportion increases with the temperature difference.

## Figures and Tables

**Table 1:** Compressor grid independence verification.

Grid count (million)	0.94	1.5	2.4	3.5	5.8
Outlet velocity (m/s)	168.2	129.1	142	183.9	183.9

**Table 2:** Setting of compressor simulation initial conditions.

Reduced speed	1.0	2.0
Expansion ratio	1	2.138
Total inlet temperature	1	1
Inlet total pressure	1	1
Outlet static pressure	1	1.1875

**Table 3:** Grid independence verification of high-pressure turbines.

Grid number (million)	0.215	0.374	0.551	0.86	1.04
Outlet velocity (m/s)	202.2	172.9	240.3	245.9	245.8

**Table 4:** Setting of initial conditions of high-pressure turbines.

Reduced speed	0.9	0.9	1.0	2.0
Expansion ratio	1	2.112	1	2.107
Total inlet temperature	1	1	1	1
Inlet total pressure	1	1	1	1
Outlet static pressure	1	0.623	0.94	0.534

**Table 5:** Grid independence verification of power turbines.

Grid number (million)	0.426	0.693	0.104	0.12	0.165
Outlet velocity (m/s)	146.2	81.5	136.2	178.5	178.5

**Table 6:** Setting of initial conditions of power turbine.

Reduced speed	0.9	0.9	1.0	1.0
Expansion ratio	1	2.05	1	2.05
Total inlet temperature	1	1	1	1
Inlet total pressure	1	1	1	1
Outlet static pressure	1	0.487	0.969	0.469

**Table 7:** Offset of the relevant parameters relative to the adiabatic boundary conditions.

Rotate Speed		Steady State	Cold State	Thermal State
	Heat exchange amount (kW)	−53.186	−129.239	14.498
1.0	Total enthalpy change (kW)	104.689	56.832	103.885
	Technical work (kW)	158.172	186.071	89.387

**Table 8:** Results of adiabatic/steady boundary conditions of power turbines under different working conditions.

Reduced speed	0.8	0.8	0.8	0.8	1.0	1.0	1.0	1.0
Boundary conditions	Adiabatic	coupling	Adiabatic	coupling	Adiabatic	coupling	Adiabatic	coupling
Total expansion ratio	1	0.9995	1.907	1.908	1.009	1.008	1.976	1.976
Reduced mass-flow	1	1	1.026	1.026	1.003	1.006	1.026	1.026
Turbine efficiency (%)	1	0.9967	1.015	1.013	0.994	0.9916	1.016	1.014

**Table 9:** Thermal Boundary Conditions of Compressor Wall Surface.

Dimensionless Heat Transfer Coefficient q*	Heat Flux Φ (J/(m^2^·s))	Direction of Heat Transfer
0	0	Adiabatic
−0.001	−860	Wall absorbs heat
+0.001	+860	Wall heat release

**Table 10:** Thermal Boundary Conditions of Turbine Wall Surface.

Dimensionless Heat Transfer Coefficient q*	Heat Flux Φ (J/(m^2^·s))	Direction of Heat Transfer
0	0	Adiabatic
−0.02	−29,360	Wall absorbs heat
−0.01	−14,680	Wall absorbs heat
+0.02	+34,060	Wall heat release
+0.05	+73,400	Wall heat release

**Table 11:** Table of values for wall thermal boundary conditions.

Dimensionless Heat Transfer Coefficient q*	Heat Flux Φ (J/(m^2^·s))	Direction of Heat Transfer
0	0	Adiabatic
−0.01	−17,030	Wall absorbs heat
+0.02	+34,060	Wall heat release

## Data Availability

The data presented in this study are available on request from the corresponding author due to privacy.
